# 27-Hydroxycholesterol Contributes to Lysosomal Membrane Permeabilization-Mediated Pyroptosis in Co-cultured SH-SY5Y Cells and C6 Cells

**DOI:** 10.3389/fnmol.2019.00014

**Published:** 2019-03-01

**Authors:** Si Chen, Cui Zhou, Huiyan Yu, Lingwei Tao, Yu An, Xiaona Zhang, Ying Wang, Yushan Wang, Rong Xiao

**Affiliations:** Beijing Key Laboratory of Environmental Toxicology, School of Public Health, Capital Medical University, Beijing, China

**Keywords:** 27-hydroxycholesterol, lysosomal membrane permeabilization, pyroptosis, cell co-culture, SH-SY5Y cells and C6 cells

## Abstract

**Purpose**: Emerging evidence suggests that 27-Hydroxycholesterol (27-OHC) causes neurodegenerative diseases through the induction of cytotoxicity and cholesterol metabolism disorder. The objective of this study is to determine the impacts of 27-OHC on lysosomal membrane permeabilization (LMP) and pyroptosis in neurons in the development of neural degenerative diseases.

**Methods**: In this study, SH-SY5Y cells and C6 cells were co-cultured *in vitro* to investigate the influence of 27-OHC on the function of lysosome, LMP and pyroptosis related factors in neuron. Lyso Tracker Red (LTR) was used to detect the changes of lysosome pH, volume and number. Acridine orange (AO) staining was also used to detect the LMP in neurons. Then the morphological changes of cells were observed by a scanning electron microscope (SEM). The content of lysosome function associated proteins [including Cathepsin B (CTSB), Cathepsin D (CTSD), lysosomal-associated membraneprotein-1 (LAMP-1), LAMP-2] and the pyroptosis associated proteins [including nod-like recepto P3 (NLRP3), gasdermin D (GSDMD), caspase-1 and interleukin (IL)-1β] were detected through Western blot.

**Results**: Results showed higher levels of lysosome function associated proteins, such as CTSB (*p* < 0.05), CTSD (*p* < 0.05), LAMP-1 (*p* < 0.01), LAMP-2; *p* < 0.01) in 27-OHC treated group than that in the control group. AO staining and LTR staining showed that 27-OHC induced lysosome dysfunction with LMP. Content of pyroptosis related factor proteins, such as GSDMD (*p* < 0.01), NLRP3 (*p* < 0.001), caspase-1 (*p* < 0.01) and IL-1β (*p* < 0.01) were increased in 27-OHC treated neurons. Additionally, CTSB was leaked through LMP into the cytosol and induced pyroptosis. Results from the present study also suggested that the CTSB is involved in activation of pyroptosis.

**Conclusion**: Our data indicate that 27-OHC contributes to the pathogenesis of cell death by inducing LMP and pyroptosis in neurons.

## Introduction

Cholesterol plays a key role in brain physiology and function. Its alterations in homeostasis and levels have been linked to neurodegeneration such as Alzheimer’s disease (AD; Arenas et al., 2017). Evidences have suggested that increased occurrence of AD is associated with raised cholesterol level (Martins et al., 2009; Di Paolo and Kim, 2011). Cerebral cells synthesize all cholesterol found in central nervous system (CNS), since the blood brain barrier (BBB) makes cholesterol’s metabolism in the brain independent to the rest of the organism (Costa et al., 2018). The question of how increased plasma cholesterol leads to the development of AD has yet to be answered.

In brain cholesterol pool, only the oxysterol metabolites of 27-hydroxycholesterol (27-OHC) and 24S-hydroxycholesterol (24S-OHC) can be exchanged with the blood circulation (Czuba et al., 2017). 27-OHC can cross the BBB from peripheral circulation to the brain with the greatest abundance. It’s concentration is comparable to or slightly higher than other oxysterols in circulation (Nelson, 2018). In fact, several studies have already described increased levels of 27-OHC in serum (Popp et al., 2012) and cerebrospinal fluid (CSF; Besga et al., 2012) of AD patients not only as a reflection of the severity of disease but also the loss of metabolically active neurons and the degree of structural atrophy (Leoni et al., 2011; Popp et al., 2013), suggesting the critical role of 27-OHC in neuro-degeneration.

Hydrolytic enzymes capable of degrading macromolecules and cell components were contained in lysosomes, which are cytoplasmic membrane-enclosed organelles. Previous studies have shown the role of lysosome dysfunction in neurodegenerative diseases (Ferguson, 2018a,b). We also found the possible involvement of plasma 27-OHC increase in disorders of lysosome function and cholesterol metabolism in brain tissues of rats (Zhang et al., 2018). These studies reveal the possible role of 27-OHC in induction of lysosome dysfunction. However, the mechanism of how 27-OHC influences lysosome function still remains uncertain.

Partial and selective lysosomal membrane permeabilization (LMP) can induce controlled cell death (Galluzzi et al., 2018). As a form of lytic programmed cell death, pyroptosis is initiated by inflammasomes (Kovacs and Miao, 2017), which is composed of a nod-like receptor (NLR) family member, procaspase-1, and the adapter protein ASC usually. The best representative of NLR family member is NLRP3, which can be activated by different stimuli, such as lysosome destabilization. Previous research has shown that pyroptosis can be induced by cathepsin B (CTSB) that leaked through LMP into the cytosole (Orlowski et al., 2017). The CTSB release from LMP could lead to activation of NLRP3 inflammasome and NLRP3-dependent neuronal pyroptosis can result in neurodegenerative diseases such as AD (Olsen and Singhrao, 2016; Li et al., 2018).

Cholesterol oxidation metabolites have been found to be one of the important factors inducing LMP (Boya, 2012). Cell experiments *in vitro* found that 7β-hydroxycholesterol (7β-OHC)and 7-ketocholesterol (7-KC) can induce cell death through LMP (Laskar et al., 2013; Yuan et al., 2016). However, whether 27-OHC, as one of the important oxysterols, can lead to LMP is still unclear.

We used the co-culture system to simulate a proper environment for the growth of neurons in the body to investigate the effect of 27-OHC. In the *in vitro* co-culture system neuron and astrocyte can support each other through the secretion of soluble factors among cells (Ma et al., 2015). In order to research the influence of 27-OHC on the function of lysosome and LMP which then induces pyroptosis in neuron, SH-SY5Y cells (human neuroblastoma cell line) and C6 cells (rat glial cell line) were co-cultured in this study.

## Materials and Methods

### Reagents and Cell Culture

27-OHC was purchased from Santa Cruz Biotechnology Company (Dallas, TX, USA). Ten milligram 27-OHC was completely dissolved in 24.83 ml of absolute ethanol to 1,000 μM as the stock solution. Then the stock solution was dispensed into a centrifuge tubes by 1 ml per tube, and blew dry with nitrogen gas. The tubes were finally preserved at −80°C. Before each cell treatment, 27-OHC was first diluted in 0.08 ml ethanol and then added to culture medium to a final concentration of 5, 10 and 20 μM, containing 0.04%, 0.08% and 0.16% ethanol (v/v).

SH-SY5Y cells (human neuroblastoma cell line) were purchased from Peking Union Medical College Cell Resource Center (CRC/PUMC) and C6 cells (rat glial cell line) were purchased from Cell Bank, Shanghai Institutes for Biological Sciences were grown in Dulbecco’s modified eagles medium (DMEM) supplemented with 10 % fetal bovine serum (FBS) and penicillin (100 U/ml)/streptomycin (100 U/ml) at 37°C in an atmosphere of CO_2_ (5%)/air (95%).

In order to simulate the environment in the brain, co-cultures of neuronal SH-SY5Y and astrocytic C6 cells were grown in a trans-well system with a 0.4 μm pore size (4.0 × 10^6^ pores/cm^2^). Neuronal SH-SY5Y cells (1.0 × 10^6^ cells) were cultured in the lower compartment of a 6-well trans-well system, while astrocytic C6 cells (5.0 × 10^5^ cells) were seeded in the insert. The insert and lower compartment are separated by polyester fiber film (Yang et al., 2005). The upper and lower compartments were cultured for 4 h separately, and then the insert was inoculated into a 6-well trans-well system. After 24 h, cells with DMEM were set as control and others were treated with 5, 10, and 20 μM 27-OHC for 24 h. 1.0 × 10^ 7^ cells were collected and analyzed finally. The choice of 27-OHC concentration in the study was referenced from previous study of our group (Wang et al., 2016; An et al., 2017).

### Cathepsin Activity Fluorometric Assay

The enzymatic activities of CTSB and cathepsin D (CTSD) in SH-SY5Y cells and C6 cells were tested using the CTSB and CTSD activity fluorometric assay kit (NO. K140-100, NO. k143-100, Biovision, Milpitas, CA, USA). Briefly, cells were collected (1 × 10^6^) by centrifugation. Lysed cells in 50 μl of cell lysis buffer and incubated cells on ice for 10 min. Centrifuge at 20,000 *g* for 5 min, and then transferred the supernatant to a new tube. Added 50 μl of cell lysate to the opaque black 96-well plate. Then 50 μl of reaction buffer and 2 μl of the 10 mM substrate Ac-RR-AFC were added to each sample. For negative control, added 2 μl of inhibitor. Enspire multifunctional microplate reader was used with a 400-nm excitation and 505-nm emission filter to analyze fluorescence intensity for CTSB enzymatic activities, and with a 328-nm excitation and 460-nm emission filter for CTSD enzymatic activities after incubating at 37°C for 2 h in the dark.

### Lyso-Tracker Red Staining

Lysosomal staining was performed using Lyso-Tracker Red (LTR). After 24 h of treatment with 27-OHC in 20-mm glass-bottom dish, cells (1.0 × 10^6^ cells/ml) were incubated with LysoTracker Red at 5 nmol/L for 30 min at 37°C and washed three times with phosphate-buffered saline (PBS). The cells were then inspected and photographed with the aid of a TCS SP8 STED confocal microscope (Leica; Germany).

### Acridine Orange Staining

After the designated treatments in 20-mm glass-bottom dish, cells (1.0 × 10^6^ cells/ml) were incubated with medium containing 5 μg/ml acridine orange (AO) for 15 min at 37°C and rinsed with PBS. Then the cells were imaged under the confocal microscope with the excitation wavelength set at 488 nm; two separate emission bands (505–570 nm and 615–754 nm) were obtained. Enspire multifunctional microplate reader was used to analyze fluorescence intensity after AO staining.

### Quantitative Analysis of the Integrity of Lysosomes

One-hundred microliter per well AO (5 μg/ml in PBS) was added to 96-well plate for labeling cells for 5 min. After rinsing with PBS three times, the fluorescence intensity was analyzed using an Enspire multifunctional microplate reader with a 475-nm excitation and 520-nm emission filter for green fluorescence.

### Scanning Electron Microscopy

Cells were fixed with 2.5% glutaraldehyde for 3 h, and then rinsed with 0.1% PB three times. Then they were dehydrated through a graded series of ethanol (30, 50, 70, 95 and 100%) and dried by the tertiary butanol method. The samples were then mounted on metal stubs and dried in a silica gel vacuum desiccator. They were sputter coated with gold and examined under a Hitachi S-4800 scanning electron microscope (SEM) operating at 15 kV.

### Subcellular Fractionation

After 27-OHC exposure, SH-SY5Y cells were washed twice with PBS and collected using a cell scraper. The lysosome fractions were separated using a Lysosome Enrichment Kit (Bestbio, Shanghai, China) according to the manufacturer’s instructions. In short, collected cells were resuspended with Lysosome Isolation Reagent A, and oscillated on ice for 10 min. Then the cells were homogenized 30–40 times in a Dounce homogenizer followed by centrifugation at 4,000 *g* for 10 min. Supernatant fractions were centrifuged at 20,000 *g* for 20 min and the supernatant was collected and labeled as cytosolic fraction. The resulting pellets were further spun at 20,000 *g* for 20 min after being resuspended with Lysosome Isolation Reagent B. The pellets were resuspended with Lysosome Isolation Reagent C (2 μl of protease inhibitor cocktail per 200 μl of reagent C) and added to the resulting pellets and oscillated on 4 for 15–30 min. The lysis was further spun at 12,000 *g* for 15 min and the supernatant was Lysosome total protein enriched fraction.

### Western Blot Analysis

Cells treated with DMEM in addition to 27-OHC with concentrations of 5 μM, 10 μM and 20 μM respectively. After the treatments, cold PBS were used to wash cells and 0.1 ml cell lysis buffer was added to superstratum and substratum of transwell plate. Cell extracts were collected by centrifugation at 13,000 *g* for 15 min at 4°C. The BCA Protein Assay Kit was used to measure the protein concentration. Equal amount of proteins (20 μg) were loaded on 8% or 10% SDS-acrylamide gels for separation by electrophoresis and then transferred onto PVDF membrane. After being blocked with 5% non-fat milk, immunoblots were probed with appropriate antibodies. The anti-CTSB (1:1,000, #31718) from Cell Signaling Technology, Danvers, MA, USA; anti-CTSD (1:1,000, #21327-1-AP) from proteintech, Rosemont, IL, USA; anti-lysosomal-associated membrane protein-1 (anti-LAMP-1; 1:1,000, #ab24170) from Abcam, USA; anti-LAMP-2 (1:1,000, #sc-71492) from Santa Cruz Biotechnologies, Dallas, TX, USA; anti-NLRP3 (1:1,000, #ab214185) from Abcam, USA; anti-gasdermin D (anti-GSDMD; 1:2,000, #orb390052) from biorbyt, UK; anti-interleukin (anti-IL)-1β; 1:1,000, #sc-52012) from Santa Cruz Biotechnologies, Dallas, TX, USA; anti-caspase-1 (1:1,000, #ab1872) from Abcam, USA were used as primary antibodies. β-Actin was used as a control of total protein. After overnight incubation with primary antibodies and washing three times with TBST, the PVDF membranes were incubated with anti-rabbit IgG or anti-mouse IgG for 1 h. The membranes were then scanned using a Vilber Lourmat Fusion SL6 (Vilber; France), and the gray values were analyzed through ImageJ software at last. Each experiment was repeated at least three times.

### Statistical Analysis

The software SPSS 19.0 (Chicago, IL, USA) was used to analyze the data. All results were represented as means ± SE of at least three independent experiments. One-way analysis of variance (ANOVA) and least significance difference (LSD) were used to evaluate statistical significance of the difference between groups.* P* values < 0.05 were considered as significant.

## Results

### 27-OHC Caused Differential Regulation of Cathepsin B and D Protein and Activity in Co-cultures of SH-SY5Y Cells and C6 Cells

**Figure 1:**
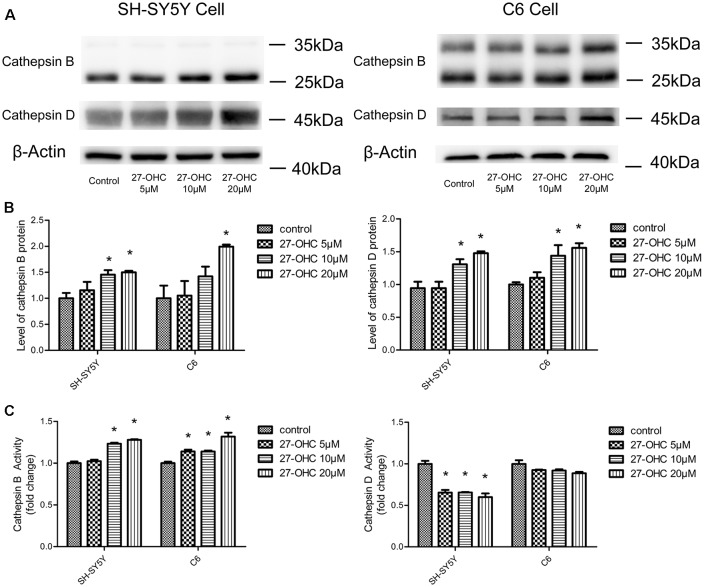
27-Hydroxycholesterol (27-OHC) treatment causes differential regulation of cathepsin B (CTSB) and cathepsin D (CTSD) protein and activity in co-cultures of SH-SY5Y cells and C6 cells. SH-SY5Y cells and C6 cells were treated with normal medium or 5 μM, 10 μM, 20 μM 27-OHC. The protein levels of CTSB and CTSD were analyzed with western blotting **(A,B)**. **(C)** The activities of CTSB and CTSD were measured by fluorometric method using commercially available kits. All data are presented as the means ± SE. *n* = 3–4 for protein and *n* = 3 for activity. *p < 0.05 compared with the control group.

To observe the effect of 27-OHC on the function of lysosome, we examined the changes in protein content and enzymatic activity of CTSB and CTSD in SH-SY5Y cells and C6 cells. The active form of CTSB (24 and 27 kDa) was detected by western blot (Nagakannan and Eftekharpour, 2017). We found that 27-OHC treatment significantly regulated the protein content of CTSB and CTSD in both SH-SY5Y cells and C6 cells. In SH-SY5Y cells, the protein levels of CTSB and CTSD were up-regulated with treatments of both 10 μM and 20 μM 27-OHC (Figures 1A,B). In C6 cells, CTSB protein level was up-regulated by treatment of 10 μM 27-OHC, and both CTSB and CTSD protein content were significantly up-regulated by treatment with 20 μM 27-OHC (*p* < 0.05). To further confirm these changes in CTSB and CTSD protein activities, the commercially available enzymatic activity assay kits (Biovision, Milpitas, CA, USA) were used. We observed robust increased enzymatic activity of CTSB in both two cell lines with 27-OHC treatment. The enzymatic activity of CTSD was reduced in SH-SY5Y cells treated with 5 μM, 10 μM, and 20 μM 27-OHC, while no significant change was observed in 27-OHC treated C6 cells (Figure 1C).

These data suggested that 27-OHC causes differential changes in protein content and activity of CTSB and CTSD in co-cultures of SH-SY5Y cells and C6 cells. The increase in CTSB levels in 27-OHC treated cells was coincided with the significant upregulation of CTSB activity. However, the new protein synthesis is independent of CTSD activity. Since previous research has shown that the change of CTSB and CTSD is a specific indicator of lysosomes dysfunction (Ashtari et al., 2016; Schultz et al., 2016), these results may indicate that 27-OHC could affect the lysosomal function of C6 and SH-SY5Y cells.

### Lysosomal Deficiency Caused by 27-OHC Treatment Was Shown as Lysosomal Membrane Permeabilization in SH-SY5Y Cells and C6 Cells

**Figure 2:**
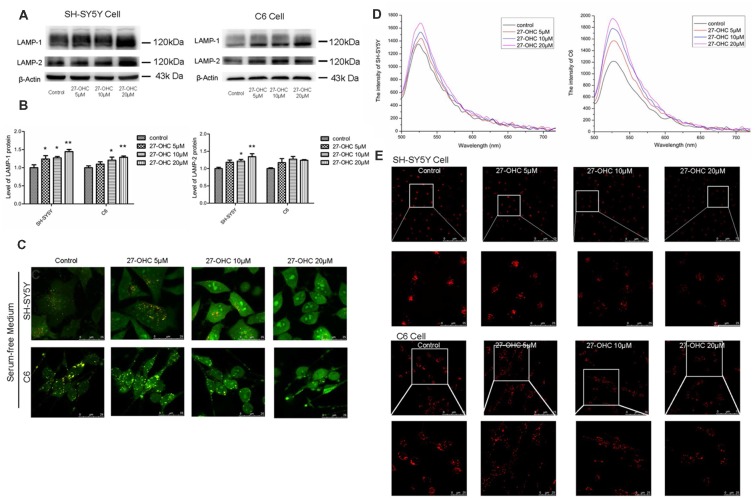
27-OHC causes lysosomal membrane permeabilization (LMP) in SH-SY5Y cells and C6 cells. **(A,B)** Representative western showed LAMP1 and LAMP2 levels in SH-SY5Y cells and C6 cells subjected to different concentrations 27-OHC (0, 5, 10, 20 μM) for 24 h. β-Actin was used as a normalized protein. **(C)** SH-SY5Y cells and C6 cells were treated with normal culture medium or 27-OHC (5, 10, 20 μM) for 24 h, stained with 5 μg/ml acridine orange (AO) for 15 min and imaged under confocal microscope (scale bar: 25 μm). **(D)** The spectrum of SH-SY5Y cells and C6 cells subjected to 27- OHC (0, 5, 10, 20 μM) for 24 h; *n* ≥ 100 cells. **(E)** Effect of 27-OHC on lysosomes. SH-SY5Y cells and C6 cells were with 27-OHC (0, 5, 10, 20 μM) for 24 h. The lysosomes were labeled by 5 nM Lyso-Tracker Red (LTR) and examined by confocal (scale bar: 75 μm and 25 μm). All the data were shown as mean ± SE; *n* = 3–4 for protein. *p < 0.05, **p < 0.01 compared with control group.

To determine whether lysosomal deficiency was associated with 27-OHC, we first tested lysosomal-associated membrane proteins LAMP-1 and LAMP-2 after 27-OHC treatment. Administration of 27-OHC treatment remarkably increased the content of LAMP-1 protein in SH-SY5Y cells and C6 cells (*p* < 0.01; Figures 2A,B) and LAMP-2 protein in SH-SY5Y cells (*p* < 0.01; Figures 2A,B). However, there was no significant difference in the content of LAMP-2 protein between 27-OHC and control treatments in C6 cells. These results indicated that in both cell lines 27-OHC treatment leads to enlargement of lysosomes (Nagakannan and Eftekharpour, 2017), which has been suggested to increase the possibility of lysosomes losing their integrity, leading to leakage of lysosomal content to the cytosol (Appelqvist et al., 2013).

To investigate whether 27-OHC treatment leading to lysosomal deficiency was associated with LMP induction, we used AO staining to detect the acid lysosomes (Hsieh et al., 2017). AO emits a granular red fluorescence in a normal lysosomal acidic environment, but a granular green fluorescence in neutral and slightly basic environment such as cytosol. Once the lysosomal integrity was destroyed, AO would relocate from lysosomes to cytosol and can then be detected by an enhanced green fluorescence, indicating the occurrence of LMP. We positioned distinct orange-red particles represents complete lysosome in control (Figure 2C). We noted decreased acidophilic lysosomes and an enhanced green fluorescence in the cytosol after treatment with 27-OHC in a dose-dependent manner in SH-SY5Y cells and C6 cells (Figure 2C). We used enspire multifunctional microplate reader to analyze fluorescence intensity after AO staining. Green fluorescence was enhanced in both SH-SY5Y and C6 cells, which indicated that LMP was happening (Figure 2D).

To further examine the changes of lysosomal function caused by 27-OHC treatment, LTR was used to label lysosomes in live cells. As an acidotropic probe, the decreased LTR fluorescence reflected both a rise in pH of lysosomal and a reduction in number of lysosomes (Li et al., 2017). Treatment with 27-OHC markedly reduced the fluorescence intensity, indicating that 27-OHC treatment reduced intracellular acidic components and the number of lysosome (Figure 2E), and the possibility of LMP occurrence in SH-SY5Y cells and C6 cells.

### 27-OHC Caused Cathepsin B Leakage Into the Cytoplasm Around the Lysosomes With LMP in SH-SY5Y Cells

**Figure 3:**
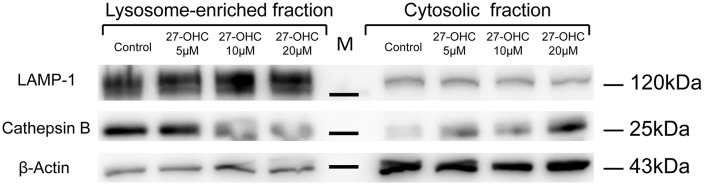
27-OHC causes CTSB leakage into the cytoplasm around the lysosomes with LMP in SH-SY5Y cells. 27-OHC treatment caused the spillage of CTSB into the cytosol. Cell fractionation was performed to separate lysosomal and cytosolic fractions in SH-SY5Y cells with 27-OHC treatment (0, 5, 10, 20 μM) in serum-free medium for 24 h as indicated. Representative western blotting showing CTSB was detected in the different fractions. β-actin and LAMP1 were used as cytosolic and lysosomal markers. *n* = 3 for experiment.

It is known that induction of LMP, translocation of CTSB from lysosomes into cytosol can cause lysosomal membrane damage and initiate pyroptosis procedures (Gorojod et al., 2015; Orlowski et al., 2017). In order to determine whether CTSB leaks into the cytosol from lysosome upon induction of LMP, we carried out subcellular fractionation of SH-SY5Y cells to directly measure the location of CTSB. Western blotting results showed that 27-OHC treatment caused the spillage of CTSB into the cytosol which in a dose-dependent manner in SH-SY5Y cells (Figure 3). The results suggested that 27-OHC treatment increased the protein levels of CTSB and lead to its leakage into the cytosol around the lysosomes.

### 27-OHC Treatment Caused Pyroptosis in SH-SY5Y Cells

**Figure 4:**
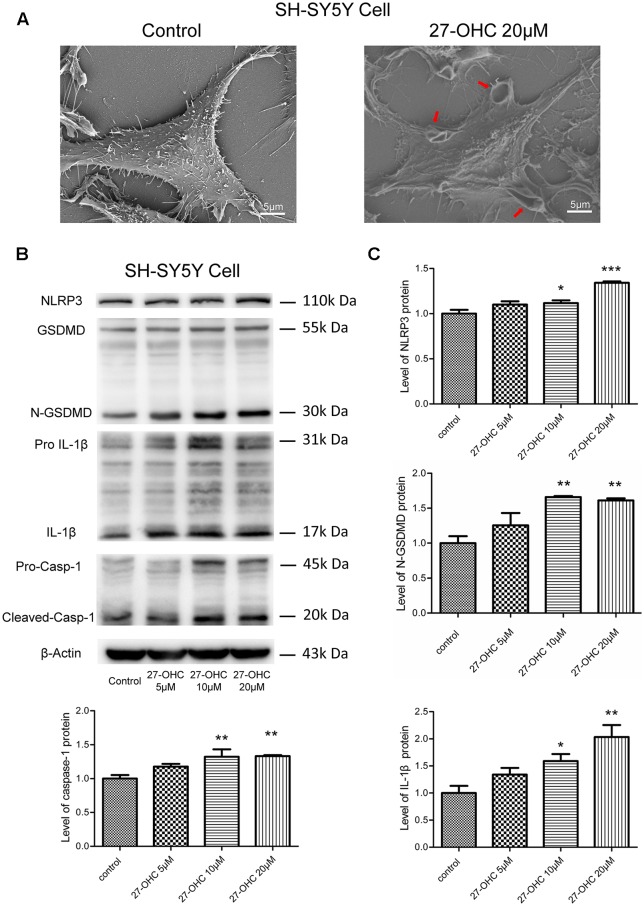
27-OHC treatment causes pyroptosis in SH-SY5Y cells. **(A)** The scanning electron microscope (SEM) showed SH-SY5Y cells swelling and membrane breakage after 27-OHC treatment, as indicated by the arrows. **(B,C)** The protein levels of nod-like recepto P3 (NLRP3), gasdermin D (GSDMD), Caspase-1, interleukin-1β (IL)-1β was analyzed by western blotting. All the data were shown as mean ± SE; *n* = 3–4. *p < 0.05, **p < 0.01, ***p < 0.001 compared with control group.

To investigate if 27-OHC treatment can lead to pyroptosis, we first analyzed morphological changes in SH-SY5Y cells after treatment with 20 μM 27-OHC by means of SEM. Through comparing the 27-OHC treatment group with the control groups, we observed swelling and membrane breakage in SH-SY5Y cells (Figure 4A), presumably due to 27-OHC treatment.

Recent findings suggested that GSDMD is the executant of pyroptosis and is in charge of the secretion of matured IL-1β (He et al., 2015). Caspase-1 can cleave GSDMD to generate an N-terminal (N-GSDMD; 30–31-kDa) and a C-terminal fragment (C-GSDMD; 22-kDa), and only the N-terminal fragment of GSDMD can induce pyroptosis (Man and Kanneganti, 2015; Shi et al., 2015). Research also proposed that the NLRP3 inflammasome can control both caspase-1-mediated pyroptosis and secretion of mature IL-1β (He et al., 2016). We thus analyzed whether 27-OHC can modulate the content of NLRP3, GSDMD, caspase-1 and IL-1β. Western blotting was conducted in SH-SY5Y cells and an increase of IL-1β protein (17-kDa) triggered by 27-OHC after 24 h of stimulation was observed (Figures 4B,C). Pro-IL-1β have been cleaved or activated after 27-OHC treatment. We also found increased NLRP3, caspase-1 and the cleaved GSDMD N-terminal fragment (N-GSDMD) at 30 kDa with 27-OHC treatment compared to the control group (Figures 4B,C). These results showed that 27-OHC may cause pyroptosis in SH-SY5Y cells.

### LMP Mediated the Activation of Pyroptosis After 27-OHC Treatment in SH-SY5Y Cells

**Figure 5:**
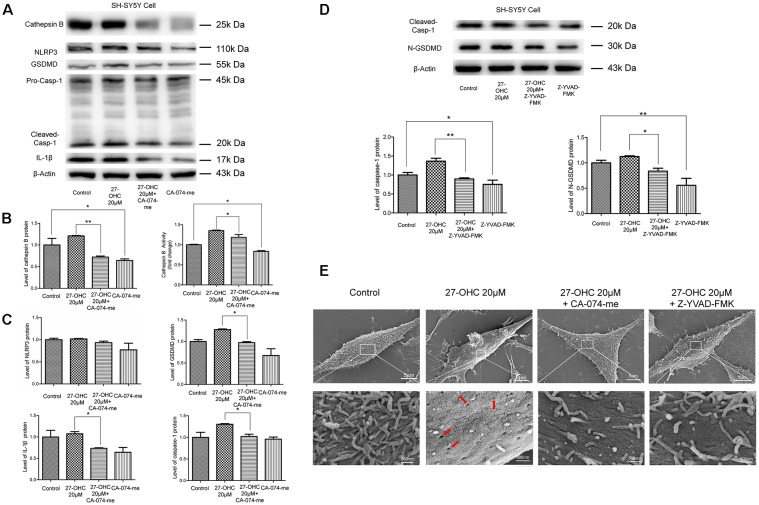
LMP mediates the activation of pyroptosis after 27-OHC treatment in SH-SY5Y cells. **(A)** SH-SY5Y cells were treated with normal culture medium, 27-OHC (20 μM), 27-OHC (20 μM) + CA-074-me (15 μM) and CA-074-me (15 μM) for 24 h, and then the cell lysates were analyzed for pyroptosis markers by western blotting. **(B)** Densitometric analysis and activity results of CTSB. **(C)** Densitometric analysis of pyroptosis-associated proteins normalized to β-Actin is shown. **(D)** SH-SY5Y cells were treated with normal culture medium, 27-OHC (20 μM), 27-OHC (20 μM) + Z-YVAD-FMK (10 μM) and Z-YVAD-FMK (10 μM) for 24 h and the cell lysates analyzed for caspase-1 and GSDMD by western blotting. **(E)** SEM showed some pores on SH-SY5Y cells membranes after 27-OHC 20 μM treatment (as indicated by the arrows) and absence/reduced pore formation after 27-OHC treatment with anti-CTSB or anti-GSDMD. All the data were shown as mean ± SE; *n* = 3–4. *p < 0.05, **p < 0.01 compared with control group.

Recent researches have proposed that LMP can result in releases of CTSB into the cytosol and then activate NLRP3 (Duewell et al., 2010; Serrano-Puebla and Boya, 2016). Therefore, we analyzed the effect of LMP and/or CTSB on protein content of pyroptosis-associated proteins. We used CTSB inhibitor (CA-074-me, 15 μM) to pretreat SH-SY5Y cells for 1 h at 37°C before 27-OHC treatment. The western blotting and activity results suggested decreased levels of CTSB whencompared to the control group (Figures 5A,B). The protein content of GSDMD, caspase-1 and IL-1β were also examined. We found down regulation of protein content of all the three pyroptosis-associated proteins after co-treating with 27-OHC and CA-074-me. There were no statistically significant differences of NLRP3 protein content between each group, but the results revealed a clear decreased tendency (Figures 5A,C). To report this point more thoroughly, we used caspase-1 inhibitor (Z-YVAD-FMK, 10 μm; Wang et al., 2015) to pretreat SH-SY5Y cells for 30 min at 37°C before 27-OHC treatment to suppress expression of GSDMD, which was proved to be effective through the results of western blot (Figure 5D). The results of SEM shown pores with size of 24–50 nm were formed on membranes after 27-OHC 20 μM treatment by 500.0 nm scale bars. Beyond that, there is no/little pore on cell membranes with CA-074me and Z-YVAD-FMK treatment in SEM (Figure 5E). The results suggested that CTSB is implicated in activation of pyroptosis. The results are consistent with previous studies indicating that CTSB released from ruptured lysosomes can activate pyroptosis (Brojatsch et al., 2015; Yang et al., 2016).

## Discussion

The amyloid hypothesis posits that β-amyloid (Aβ) is the pathological factor that initiates the onset and progression of AD (Tam et al., 2016). Earlier studies have shown that amyloid precursor protein (APP) is the source of Aβ. And many studies indicated that nascent APP is cleaved after endocytosis from the cell surface into endosomes and subsequently into lysosomes. Recent research has demonstrated that APP degraded by lysosomal system (Hein et al., 2017). CTSB is a most abundant cysteine protease with potentially specific roles that cleaves APP and also degrades the Aβ42 peptide (Llorente et al., 2018) in the endo-lysosome system. Lysosomes are ubiquitous organelles that constitute the primary degradative compartments of the cell. They receive their substrates through endocytosis, phagocytosis or autophagy (Saftig and Klumperman, 2009). Thus, impairment of lysosomal function plays an important role in neuronal degeneration and in the pathogenesis of numerous neurodegenerative diseases (Cermak et al., 2016). Studies demonstrated that abnormalities of the endosomal-lysosomal system are also early pathological features in the AD brain (Barbero-Camps et al., 2018).

This study found that 27-OHC changed the levels of lysosome protein, CTSB and CTSD, and induced lysosome dysfunction in co-cultured SH-SY5Y cells and C6 cells. It revealed for the first time the effects of 27-OHC treatment on CTSB and CTSD *in vitro*. The effects of oxysteroids on structure and function of lysosomes have been proposed in a previous study (Boya, 2012). Excessive accumulation of oxysteroids in cells can lead to leakage of lysosomal membrane (Laskar et al., 2013; Yuan et al., 2016). However, as one of the important oxidation products of cholesterol, how 27-OHC influences the structure and function of lysosomes remains unknown. In our previous research, we found that increased level of plasma 27-OHC might affect the cholesterol metabolism and lysosome function *in vivo* (Zhang et al., 2018) and our previous study has demonstrated that excessive 27-OHC with concentrations of 7, 21 and 70 μM in blood can impair spatial learning and memory and contributes to the perturbation of cholesterol metabolism in the rat brain (Zhang et al., 2015). Further studies on co-culture neurons have shown that 27-OHC can induce cytotoxicity and present a dose-effect and time-effect correlation (Wang et al., 2016). Wang found 5 μM, 10 μM, 20 μM of 27-OHC treatment caused significant inhibition of cell viability and the loss of mitochondrial membrane potential (so the concentrations used 5 μM, 10 μM, 20 μM of 27-OHC in our study). The present study found that 27-OHC treatment can up-regulate protein levels of CTSB and CTSD and activity of CTSB in both of SH-SY5Y cells and C6 cells in co-culture conditions. Up-regulation of CTSB and CTSD is a specific indicator of lysosomes dysfunction (Ashtari et al., 2016; Schultz et al., 2016). So the data obtained here confirmed that 27-OHC treatment can cause lysosomal dysfunction.

27-OHC treatment increased the protein content and accumulation of LAMP-1 and LAMP-2, which indicated the larger volume of lysosome (Nagakannan and Eftekharpour, 2017). The larger lysosomes are more prone to undergo membrane damage as had been verified in a previous study (Ono et al., 2003). In this research, we report that 27-OHC treatment can destroy the lysosomal membrane of SH-SY5Y cells and C6 cells accompany with the increased content of CTSB and CTSD and increased enzymatic activity of CTSB. For the result of reduced activity of CTSD, previous research has shown that CTSD will lose activity due to de-protonation of the residues in the active site at neutral pH, but remain stable structurally (Repnik et al., 2014).The results of AO and LTR have proved the induction of LMP by 27-OHC treatment (Figures 2C–E). The immediate consequence of LMP is leakage of lysosomal hydrolytic enzymes, which implies the release of CTSB and other lysosomal constituents from lysosome (Boya and Kroemer, 2008). And the detection results of protein content revealed that CTSB has leaked into the cytosol (Figure 3). Significantly, LTR showed more diffusion with the increasing concentration of 27-OHC-treatment, and such a distribution proved the burst of lysosome vesicles.

The previous study of our team found that 27-OHC treatment can cause cell death in co-cultured SH-SY5Y cells and C6 cells (Wang et al., 2016). In the present study, we confirmed that 27-OHC treatment can cause lysosome damage and release of CTSB from lysosomes. Some research suggested that when LMP approaches a certain level, the nerve cells will change from the stable state to cell death state (Lipton, 2013; Ni et al., 2018). Research has shown that CTSB can remain active in neutral environment after LMP even if most hydrolytic enzymes are inactive under pH-neutral conditions (Boya, 2012).

Pyroptosis has been explored by many researchers recently. Some substances can induce cleavage and secretion of IL-1 family cytokines by stimulating the caspase-1-activating NLRP3 inflammasome, which results in pyroptosis. As the best-characterized inflammasome, studies found that NLRP3 was activated in AD (Saresella et al., 2016), which can be activated by cholesterol, cathepsin and also by the amyloid peptide (Duewell et al., 2010; Masters and O’Neill, 2011; Jo et al., 2016). The idea that cathepsins play an important role in the pathogenesis of neurodegenerative disorders has been long known in the scientific literature. Their changes in concentration, activity and localization are normally found in aging neurons and are considered as a cause of age-related neuropathologic changes (Cermak et al., 2016). Orlowski found due to LMP that active CTSB released into the cytoplasm can initiate pyroptosis procedures and lead to primary murine macrophages death eventually (Orlowski et al., 2017). However, whether LMP mediated the activation of pyroptosis after 27-OHC treatment in neurons was seldom reported. Therefore, we detected pyroptosis associated proteins including NLRP3, GSDMD, caspase-1 and IL-1β in the present study. The IL-1β is a well-known unconventionally secreted leaderless proteins with important extracellular functions (Rubartelli et al., 1990). We found that 27-OHC treatment caused pyroptosis in SH-SY5Y cells in co-culture system (Figure 4). In order to investigate the role of CTSB and/or LMP in pyroptosis in SH-SY5Y cell line, CA-074-me, an inhibitor of CTSB, was used to prohibit the activity of CTSB. We demonstrated that pyroptosis was partially prevented by Ca-074-me, indicating that CTSB is involved in activation of pyroptosis (Figure 5). In general, our results showed that LMP plays a crucial role in 27-OHC-induced pyroptosis and consequently in CTSB mediated GSDMD cleavage and the resulting activation of the caspase-1 pathway. Meanwhile our results highlight the critical role of 27-OHC in pyroptosis of SH-SY5Y cells.

## Conclusion

**Figure 6:**
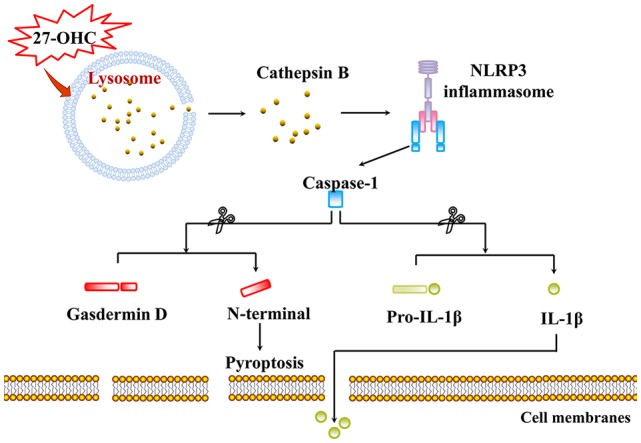
Proposed model for pyroptosis and LMP induced by 27-OHC. 27-OHC caused active CTSB to leak through LMP into the cytosol and then NLRP3 inflammasome activating. In the case, caspase-1 got activated and cleaved both GSDMD to generate a N-GSDMD and a C-GSDMD and pro-IL-1β to initiate pyroptosis and maturation of IL-1β, respectively.

We identified the role of 27-OHC in lysosome dysfunction in this research. Our data demonstrated that 27-OHC can induce LMP. We also found that CTSB leaked through LMP into the cytosol and induced NLRP3-dependent neuronal pyroptosis (Figure 6). Both references of the potential toxic effects of 27-OHC on lysosomes and their contribution to the pathogenesis of neurodegenerative diseases were provided in this study. Population-based studies and animal experiments are needed in further research to clarify the relationship between 27-OHC, lysosome dysfunction and neurodegenerative diseases. To our knowledge, this is the first study indicating the lysosome dysfunction in 27-OHC treated neurons. We speculate that 27-OHC may cause pyroptosis through LMP of nerve cells.

## Author Contributions

SC, CZ and RX designed the research. SC conducted the research and analyzed the data. SC and CZ wrote the manuscript. MJ and LJ gave technical support of SEM. XT provided linguistic assistance during the preparation of this manuscript. HY, LT, YA, XZ, YuW and YiW gave auxiliary support, and RX had primary responsibility for the final content. All authors read and approved the final manuscript.

## Conflict of Interest Statement

The authors declare that the research was conducted in the absence of any commercial or financial relationships that could be construed as a potential conflict of interest.

## Abbreviations

Aβ: β-amyloid
AO: acridine orange
AD: Alzheimer’s disease
LMp: lysosomal membrane permeabilization
24S-OHC: 24-hydroxycholesterol
27-OHC: 27-hydroxycholesterol
7-KC: 7-ketocholesterol
7β-OHC: 7β-hydroxycholesterol
BBB: blood brain barrier
CTSB: cathepsin B
CTSD: cathepsin D
CNS: central nervous system
FBS: fetal bovine serum
GSDMD: gasdermin D
LTR: Lyso-Tracker Red
NLR: nod-like receptor.
